# How the Most Trusted Venues for Health-Related Information Influence Physician Referrals to Smoking Cessation Services

**DOI:** 10.5402/2012/256301

**Published:** 2012

**Authors:** Judy Kruger, Angela Trosclair, Crystal Bruce, Diane Beistle

**Affiliations:** Office on Smoking and Health, Centers for Disease Control and Prevention, 4770 Buford Highway NE, K-50, Atlanta, GA 30341-3717, USA

## Abstract

Public health programs seek to educate physicians by using a variety of venues. Therefore, it is important to understand which health information sources physicians are using and how these sources affect referrals. We explored how venues for health-related information affect physicians’ referral practices to smoking cessation services. The 2008 DocStyles survey asked physicians to rank a list of their most trusted sources of health-related information. The analysis was restricted to 1,617 physicians who responded to all variables of interest. In this sample, the most trusted sources of health-related information cited by physicians were medical journals (95.9%), government health agencies (82.2%), other physicians (76.4%), professional medical societies (75.2%), and medical Web sites or podcasts (65.9%). Medical providers were more likely to refer tobacco users to cessation services if they used professional medical societies as a source to obtain patient health-related information, compared with medical providers not using this source (multivariate odds ratio = 1.31; 95% confidence interval = 1.03–1.66). Physicians use many health information sources. Therefore, to reach physicians effectively, a broad dissemination of guidelines and best practices in tobacco control is needed and should include information for medical societies.

## 1. Introduction

Tobacco use remains the single most preventable cause of disease and death in the United States. In 2010, 19.3% of the adult population reported smoking every day or some days [1]. Physicians are one of the most important sources of information about smoking cessation for patients who use tobacco [2]. Approximately 84% of the US population visit a primary care provider each year, and they average 2.1 visits a year, which provide ample opportunity for physicians and their staff to deliver brief, effective, tobacco cessation interventions to their patients who use tobacco [3]. Physician advice increases the number of patients who attempt to quit smoking and succeed [4, 5].

During the office medical visit or at the patient’s bedside, physicians are frontline educators and treatment providers, and the U.S. Public Health Service (PHS) guidelines confirm the physician’s role as essential for providing tobacco dependence services [3]. The PHS clinical practice guidelines for tobacco use and dependence recommend that physicians follow the 5 A’s when helping patients quit: ask, advise, assess, assist, and arrange followup [3]. Unfortunately, physicians do not routinely follow all of these guidelines. A report about physician behavior related to smoking cessation by the Association of American Medical Colleges (AAMC) includes physicians in the specialty fields of family medicine, general internal medicine, obstetrics/gynecology, and psychiatry. The AAMC report states the following: 84% of these physicians report that they routinely ask patients about smoking status; 86% advise smokers to stop smoking; 63% assess patients’ willingness to quit; 13% usually refer smokers to others for appropriate treatment; 17% usually arrange for follow-up visits to address smoking [2]. In addition, the report found that among all specialty groups, few physicians discussed counseling options, enlisted support for quitting, or monitored patient progress in cessation attempts. Physicians reported barriers to providing help, including inadequate knowledge and skills for delivering smoking cessation interventions because of limited medical education and training [2]. In addition, physicians felt that their smoking cessation activities were inadequate because of insufficient organizational resources to help patients quit, including a lack of pamphlets for waiting rooms, and onsite group, or individual counseling programs [2]. Physicians frequently mention this lack of knowledge and training for smoking cessation as a barrier to providing effective tobacco cessation treatments [3, 6]. Additional barriers to counseling patients that physicians have identified are a lack of patient interest, a lack of time allowed during patient appointments, and insufficient compensation [7].

For the types of resources that physicians are using for information, there has been an increase in electronic sources (e.g., medical outcomes software, proprietary access services, the Internet, and CD-ROM educational materials) [8–10]. Medical journal information is now available in both print and on the Web, and physicians are using the Internet to seek answers to their health-related questions [11]. Technology has the potential to provide health care providers with easy access to health information when needed.

It is important to understand which health information venues physicians are using and how these outlets affect physician referral patterns. This understanding can help public health programs focus on tobacco control, target their information to the most appropriate media outlet, and increase use of effective cessation strategies. How physicians are using health-related information venues may guide communication efforts and identify communication strategies for dissemination of tobacco cessation information. The objective of this paper is to explore the effect of health-related information venues on physicians’ smoking cessation referral practices.

## 2. Methods

### 2.1. DocStyles Survey

We selected our study population from respondents to the 2008 DocStyles survey, which was conducted by Porter Novelli, a social marketing and public relations firm. DocStyles is an annual web survey that provides insight into physicians’ attitudes, behaviors, knowledge, and counseling behaviors on health issues, and the survey assessed their use of and trust in available health information sources. The sampling was conducted by Epocrates, Inc., by using respondents identified from the Epocrates Honors Panel, an opt-in, verified panel of 135,000 medical practitioners. The primary recruitment method was based on health care providers’ self-selection to join the panel and complete the online health care survey at http://www.epocrates.com/honors, after receiving an initial email from Epocrates.

Eligible physicians were verified by checking each by first name, last name, date of birth, medical school, and graduation date against the American Medical Association’s (AMA) master file at the time of panel registration. Physicians were screened to include only those who practice in the United States; actively see patients; work in an individual, group, or hospital practice; have been practicing medicine for at least 3 years. Epocrates randomly selected a sample of eligible physicians from their main database to load into their invitation database. The sample was drawn to match AMA master file proportions for age, gender, and region. Physicians were paid an honorarium of $50–$75 for completing the survey. Respondents were not required to participate in the 140-question survey, which had multiple subparts designed to provide insights into physicians’ counseling behaviors, and were able to exit the survey at any point. For Epocrates to reach their quotas, 14,346 physicians were invited to participate. The final sample consisted of 1,880 respondents, which resulted in a response rate of 22% after adjusting for individuals who were accepted but then excluded because the quota was filled.

### 2.2. Measures

#### 2.2.1. Demographics and Source of Health-Related Information

Demographic characteristics used in the analysis included gender, age (18–35, 36–45, 46–55, or ≥56 years), and race/ethnicity (non-Hispanic white, non-Hispanic black, Hispanic, non-Hispanic Asian, other). Smoking status was dichotomized into smokers (1–7 days/week) and nonsmokers (0 days/week). Participants were asked about the frequency with which they referred patients to cessation programs: *How often, if ever, do you refer your patients who smoke tobacco or use other tobacco products to cessation programs such as a telephone quitline, a smoking cessation class, or one-on-one counseling*? Response options included *never*, *rarely*, *sometimes*, *usually*, and *always*, and these responses were dichotomized as *always/usually/sometimes* or *never/rarely*.

The 2008 DocStyles survey included a series of questions about respondents’ use of health-related information. Respondents were asked a screening question for 16 different health information sources: (1) advertising; (2) books; (3) brochures, flyers, pamphlets, and posters; (4) government health agencies; (5) pharmaceutical companies; (6) medical Web sites or medical podcasts; (7) magazine stories or articles; (8) medical journals; (9) newspaper stories or articles; (10) nurses, nurse practitioners, and physician assistants; (11) other physicians; (12) patient advocacy groups; (13) patients or parents who have researched a condition; (14) professional medical societies; (15) radio; (16) television. The question was as follows: *please indicate how often you use each of the following sources to obtain patient health-related information*. Response options were *never*, *rarely*, *sometimes*, *often*, and *regularly*. If they did not answer *never* to all sources, then they were asked to select five of their most trusted patient health-related information sources from the list of 16 health information sources.

### 2.3. Statistical Analyses

Descriptive analyses were conducted to characterize physicians by their gender, age, race/ethnicity, smoking status, and referral of patients who use tobacco to cessation services. We used a chi-square test to examine within-group differences in the proportion of physicians who stated that they *always/usually/sometimes* referred tobacco users to cessation services, compared with those who *never/rarely* referred to cessation services. To describe the most trusted sources of medical information among physicians, we stratified respondents by gender, age, and race/ethnicity to examine the five most trusted sources of health-related information. Separate logistic regression models were used to test the relationship between use of one of the five most trusted health-related information sources and referral to smoking cessation services, including gender, age, and race/ethnicity as covariates. Odds ratios with 95% confidence intervals (CIs) were calculated to compare referral to cessation services among those who reported the source as a trusted source versus those who did not select the source as one of their most trusted sources. All of the analyses were completed by using SAS version 9.2 (SAS Institute Inc., Cary, NC) software.

## 3. Results

Of the physicians who responded to the survey and all the variables of interest, 68.9% were men and 31.1% were women (Table 1). Approximately 40% (39.4%) were aged 36–45 years, and 72.9% were white. More than 93% (93.4%) were nonsmokers, and 42.7% always, usually, or sometimes referred tobacco users to cessation services.

Table 2 shows the proportion of physicians who referred their tobacco-using patients to cessation services. A significant difference in the distribution for referring versus not referring was found for sex and race/ethnicity (*P < .*05). Statistically significant differences were also found by age among physicians who always, usually, or sometimes referred tobacco users to cessation services compared with those who never or rarely referred tobacco users (*P < .*001).

In this sample, the most trusted sources of health-related information cited by physicians (in rank order) were as follows: medical journals (95.6%), government health agencies (82.7%), other physicians (76.4%), professional medical societies (75.2%), and medical Web sites or pod-casts (65.9%). Table 3 presents frequencies by gender, age, and race/ethnicity among physicians and showed that a higher proportion of female physicians than male physicians reported using government health agency information and medical Web sites or podcasts (*P < .*001). A higher proportion of male physicians (78.0%) compared to female physicians (69.0%) reported using professional medical societies as a source of health-related information (*P < .*001). Hispanic and non-Hispanic Asian physicians used government health agency information less than their non-Hispanic black and non-Hispanic white counterparts (*P < .*05). A lower proportion of non-Hispanic Asian physicians (69.8%) compared to non-Hispanic white physicians (77.3%) reported using professional medical societies (*P < .*05). Non-Hispanic Asian physicians used medical Web sites or podcasts more than non-Hispanic white (63.7%) and non-Hispanic black (66.7%) physicians (*P < .*05).

Logistic regression analyses indicated that physician use of selected health-related information sources had an increased likelihood of referring patients to smoking cessation services (Table 4). In the unadjusted model, physicians who used professional medical societies were more likely to refer smokers to smoking cessation services than those who did not (OR1.29; 95% CI = 1.02–1.63). Physicians who used medical Web sites or podcasts were more likely to refer tobacco users to cessation services than those who did not (OR = 1.25; 95% CI = 1.01–1.54). When adjusting for sex, age, and race/ethnicity, physicians were more likely refer tobacco users to cessation services if they used professional medical societies as a source to obtain patient health-related information, compared with medical providers not using this source (AOR = 1.31; 95% CI = 1.03–1.66).

## 4. Discussion and Summary

It has been recommended as standard medical practice for physicians to engage in the 5 A’s model for treating tobacco use and dependence: ask, advise, assess, assist, and arrange [3, 5]. However, referral to cessation services is only a part of the 5 A’s. Prior research has shown that physician advice for cessation increases quitting among their patients [3, 4, 12]. One study found that 42.7% of physicians referred their tobacco-using patients to cessation services. Because physicians supply highly valued advice and can potentially reach many patients multiple times, information about what influences referral patterns may lead to patient behavior change. Tobacco-using patients especially can maximize the benefits of cessation services because free telephone quitline support services are available to help individuals who want to stop smoking or using tobacco.

Physicians used multiple venues to obtain health-related information, and referrals to cessation services were related to their most trusted sources of health-related information used in this sample. Our study found that physicians who obtained their most trusted health-related information from professional medical societies had an increased likelihood of referring patients to smoking cessation services. Several medical organizations such the American Academy of Pediatrics [13], the American College of Obstetricians and Gynecologist [14], the American Medical Association [15], and the American Academy of Family Physicians [16] recommend routinely assessing tobacco use and referral of patients to cessation services. Because of the influence that professional medical societies and their recommendations have on physicians’ referral practices, specialty societies should continue to reach physicians with cessation information through multiple avenues simultaneously. Health communicators wishing to reach physicians with cessation information should consider these professional medical associations when developing their communication plans.

Electronic sources are growing in popularity and are currently used by physicians 53% of the time to seek answers to their health questions, including cessation advice [17]. In addition, physicians are turning to the Internet to access Continuing Medical Education (CME) online health information [18]. Findings from our study suggest that medical Web sites or podcasts are a trusted source of health-related information and may influence referral to smoking cessation services. In a review of physician information-seeking behaviors by Wilson and colleagues, the authors found general medical podcasts to be a valuable resource for providing an overview of the latest research on a particular topic, as well as opportunities to explore areas outside of a physician’s core discipline [10]. Further research on Internet tools designed for physicians such as medical Web sites and podcasts can help health communicators develop best practice models and disseminate health-related information more effectively.

Furthermore, because newer sources of information found on the Internet continue to grow, there is a need to enhance surveillance of new or novel technology and to train and prepare future physicians to recognize the difference between reliable and unreliable health-related sources [17]. The literature shows that health care providers are starting to use personal digital assistants (PDAs) and mobile phone text messaging systems to obtain information [18, 19], e-mail patient reminders, and follow up on treatment plans [20] and turning to other, less research-based Web-messaging tools [21], such as blogs, Twitter, Facebook, and other social network Web sites (also known as Web 2.0) for cessation advice. With the expansion of alternative, credible, health-related information outlets that are used by physicians, their influence on physician referral patterns and other cessation-related activities should be explored.

The findings reported in this study may be subject to several limitations. First, the sampling method that was used for the survey was drawn from a self-selected group (Epocrates Honor Panel) and, thus, resulted in a convenience sample. Although the method used quotas and weighting to produce a data set that matched the specialty breakdown of the AMA membership, the findings may not be representative of all US physicians, especially those who were not members of the AMA. Second, 2008 response rates were lower than previous years, which may affect the representativeness of participants. This lower response rate may be a result of the survey being almost twice as long as in previous years, and potential participants were informed of the survey length in the invitational email, which may have dissuaded participation. Third, the questions used to ascertain provider referral to cessation services have not been validated and may not accurately capture providers’ actual behavior. Finally, the sample size for some of the racial/ethnic groups was too small to generate a stable estimate and women were underrepresented in the study.

Physicians are essential frontline treatment providers for smoking cessation services, and they obtain medical information from a variety of trusted venues: medical journals, government health agencies, physician colleagues, professional medical associations, and medical Web sites or pod-casts. More research is needed about what influences referral patterns to examine why those who depend on professional medical associations for health information are more likely to refer patients to cessation services than those who do not list medical associations as a trusted source of information. Future studies are needed to expand the list of health-related information venues and include novel or new information technology outlets that physicians use and trust.

Several medical organizations have initiatives to promulgate the 5 A’s. Groups that work to disseminate guidelines and best practices in tobacco control should consider working with specialty societies to disseminate their messages. Because the health-related information sources that physicians use can affect their referral patterns, health communicators wishing to reach physicians with quitline cessation information should consider using multiple venues when developing their communication plans.

## Figures and Tables

**Table 1:** Selected characteristics of physicians—DocStyles Survey, 2008.

Characteristics	*N*	%	95% CI
Gender			
Men	1,114	68.9	66.6–71.1
Women	503	31.1	28.9–33.4
Age (y)			
18–35	327	20.2	18.3–22.3
36–45	637	39.4	37.0–41.8
46–55	452	28.0	25.8–30.2
56+	201	12.4	10.9–14.1
Race/ethnicity			
Non-Hispanic white	1,179	72.9	70.7–75.0
Non-Hispanic black	48	3.0	2.2–3.9
Hispanic	69	4.3	3.4–5.4
Non-Hispanic asian	248	15.3	13.7–17.2
Non-Hispanic other	73	4.5	3.6–5.6
Smoking status^a^			
Smoker	107	6.6	5.5–7.9
Nonsmoker	1,510	93.4	92.1–94.5
Refers to smoking cessation services^b^			
Always/usually/sometimes	690	42.7	40.3–45.1
Never/rarely	927	57.3	54.9–59.7

Total	1,617	—	—

a Smoking status was dichotomized into smokers (1–7 days/week) and nonsmokers (0 days/week).

b Cessation services consist of telephone quitline, a smoking cessation class, or one-on-one counseling.

**Table 2:** Percentage of physicians who refer their tobacco-using patients to cessation services^a^—DocStyles Survey, 2008.

	Refers to cessation services^a^		
	*N*	%	95% CI
Gender^c^			
Male	474	42.5	(39.7–45.5)
Female	216	42.9	(38.7–47.3)
Age (y)^d^			
18–35	128	39.1	(34.0–44.5)
36–45	291	45.7	(41.8–49.6)
46–55	200	44.2	(39.7–48.9)
56+	71	35.3	(29.0–42.2)
Race/ethnicity^c^			
Non-Hispanic white	485	41.1	(38.4–44.0)
Non-Hispanic black	20^b^	41.7	(28.7–55.9)
Hispanic	35^b^	50.7	(39.1–62.3)
Non-Hispanic asian	113	45.6	(39.5–51.8)
Non-Hispanic other	37^b^	50.7	(39.4–61.9)

Total	690	42.7	(40.3–45.1)

a Cessation services consist of telephone quitline, a smoking cessation class, or one-on-one counseling. Always, usually or sometimes refers to cessation services.

b Interpretation may be limited as sample size is *<*50.

c Significant at *P < .*05.

d Significant at *P < .*001, within group comparisons of physicians who always, usually, or sometimes referred tobacco users to cessation services compared to those who never/rarely referred.

**Table 3:** Physicians use of health-related information venue^a^—DocStyles Survey, 2008.

Characteristics	Venue														
	Medical journals			Government health agency			Other physicians			Professional medical societies			Medical web sites/podcasts		
	*N*	%	95% CI	*N*	%	95% CI	*N*	%	95% CI	*N*	%	95% CI	*N*	%	95% CI
Gender															
Men	1,065	95.6	94.2–96.7	898	80.6^d^	78.2–82.8	842	75.6	73.0–78.0	869	78.0^d^	75.5–80.3	699	62.7^d^	59.9–65.6
Women	481	95.6	93.4–97.1	439	87.3	84.1–89.9	393	78.1	74.3–81.5	347	69.0	64.8–72.9	366	72.8	68.7–76.5
Age (y)															
18–35	320	97.9	95.6–99.0	269	82.3	77.7–86.0	247	75.5	70.6–79.9	239	73.1	68.0–77.6	217	66.4	61.1–71.3
36–45	602	94.5^c^	92.4–96.0	522	81.9	78.8–84.7	486	76.3	72.8–79.4	484	76.0	72.5–79.1	442	69.4	65.7–72.8
46–55	430	95.1^c^	92.7–96.8	380	84.1	80.4–87.2	347	76.8	72.6–80.4	338	74.8	70.6–78.6	284	62.8^c^	58.3–67.2
56+	194	96.5	92.9–98.3	166	82.6	76.7–87.2	155	77.1	70.8–82.4	155	77.1	70.8–82.4	122	60.7c	53.8–67.2
Race/ethnicity															
Non-Hispanic white	1,135	96.3	95.9–97.2	990	84.0	81.8–86.0	918	77.9	75.4–80.1	911	77.3	74.8–79.4	751	63.7	60.9–66.4
Non-Hispanic black	45	93.8	82.3–98.0	43	89.6	77.3–95.6	31^b^	64.6	50.2–76.7	34^b^	70.8	56.6–81.9	32^b^	66.7	52.3–78.5
Hispanic	62	89.9	80.2–95.1	51	73.9^c^	62.3–82.9	52	75.4	63.9–84.1	46^b^	66.7	54.8–76.7	50	72.5	60.8–81.7
Non-Hispanic Asian	236	95.2	91.7–97.2	195	78.6^c^	73.1–83.3	180	72.6	66.7–77.8	173	69.8^c^	63.8–75.2	186	75.0^c^	69.2–80.0
Non-Hispanic other	68	93.2	84.6–97.1	58	79.5	68.7–87.2	54	74.0	62.7–82.7	52	71.2	59.9–80.4	46^b^	63.0	51.4–73.3

Total	1,546	95.6	94.5–96.5	1,337	82.7	80.8–89.9	1,235	76.4	74.2–78.4	1,216	75.2	73.0–72.0	1,065	65.9	63.5–68.1

a Physicians reported using health-related information venues: medical journals, government health agencies, other physicians, professional medical societies, and medical web sites or podcasts sometimes, often, or regularly (from a list of 16).

b Interpretation may be limited as sample size is *<*50.

c Significant at *P < .*05.

d Significant at *P < .*001.

**Table 4:** Logistic regression of physician referral to cessation services^a^ by most trusted health-related information^b^.

Use of most trusted health-related information	Unadjusted OR^c^	95% CI	Adjusted OR^d^	95% CI
Medical journals				
No	Reference		Reference	
Yes	1.15	0.71–1.87	1.24	(0.76–2.03)
Government health agencies				
No	Reference		Reference	
Yes	0.94	0.71–1.22	0.96	(0.74–1.24)
Other physicians				
No	Reference		Reference	
Yes	1.20	0.95–1.51	1.23	(0.97–1.55)
Professional medical societies				
No	Reference		Reference	
Yes	1.29	1.02–1.63^e^	1.31	(1.03–1.66)^e^
Medical Web sites/podcasts				
No	Reference		Reference	
Yes	1.25	1.01–1.54^e^	1.22	(0.99–1.51)

a Cessation services consist of telephone quitline, a smoking cessation class, or one-on-one counseling.

b Physicians reported using the 5 most trusted health-related information venues from a list of 16.

c OR refers to odds ratio. Separate logistic regression models were used to test the relationship between use of each most trusted health-related information source and referral to cessation services.

d OR is adjusted for sex, age, and race/ethnicity.

e Significant at *P < .*05.
